# Multi-modal liquid biopsy platform for cancer screening: screening both cancer-associated rare cells and cancer cell-derived vesicles on the fabric filters for a reliable liquid biopsy analysis

**DOI:** 10.1186/s40580-019-0204-3

**Published:** 2019-11-15

**Authors:** Jiyoon Bu, Jae-Eul Shim, Tae Hee Lee, Young-Ho Cho

**Affiliations:** 1 Cell Bench Research Center, Korea Advanced Institute of Science and Technology (KAIST), 291 Daehak-ro, Yuseong-gu, Daejeon, 34141 Republic of Korea; 2 Present Address: Pharmaceutical Sciences Division, School of Pharmacy, University of Wisconsin-Madison, 777 Highland Ave, Madison, Wisconsin 53705 USA; 3 Present Address: Department of Senior Healthcare, BK21 Plus Program, Graduated School, Eulji University, 77, Gyeryong-ro 771beon-gil, Jung-gu, Daejeon, Republic of Korea

**Keywords:** Circulating tumor cells, Extracellular vesicles, Cytochalasin B, Polyester fabric, Liquid biopsy, Multi-modal screening

## Abstract

Circulating tumor cells (CTCs) are receiving a great amount of scientific interest as a diagnostic biomarker for various types of cancer. Despite the recent progress in the development of highly sensitive CTC isolation devices, post-capture analysis of CTCs is still hindered by technical challenges associated with their rarity. Herein, we present a multi-modal CTC screening platform which is capable to analyze CTCs and CTC-derived extracellular vesicles (EVs), simultaneously from a single sample. Cytochalasin B (CB) treatment promotes cells to release large number of EVs from their surface, as demonstrated by CB-treated cells (5 µg/mL for 3 h) secreting 3.5-fold more EVs, compared to the non-treated cells. CB further generates 1.7-fold more EVs from the cells captured on our CTC filtration device (the fabric filter), compared to those from the cell culture flasks, owing to its multiple pore structure design which reduces the non-specific binding of EVs. Both CB-treated cancer cells and CB-induced EVs are found to overexpress tumor-associated markers, demonstrating a potential for the development of CTC dual-screening platform. Collectively, the results presented in this study reveal that our multi-modal cancer screening platform can synergistically improve the reliability and efficacy of the current CTC analysis systems.

## Introduction

Image-guided tissue biopsy is utilized as the standard diagnostic test for cancer [1]. This traditional biopsy technique has facilitated histological and molecular analysis of tumors, improving the clinical outcomes [2]. However, the results obtained from these biopsy tests often show inconsistent benefits since tumors grow, mutate, and become heterogeneous [2, 3]. Alternatively, liquid biopsy has been highlighted as an innovative tool for cancer research, which allows non-invasive, repetitive, and routine monitoring for various types of tumor. Liquid biopsy refers to all kinds of procedures that detect or quantitatively measure disease-related biomarkers from a human body fluid, mainly from blood [4]. Unlike traditional tissue biopsy techniques, liquid biopsy enables real-time monitoring of the abnormal tissues, providing more detailed information of ongoing tumor progression and therapeutic responses.

Different biomarkers have been employed for the diagnosis and prognosis of tumor. Tumor-associated antigens are one of the most well-established biomarkers that are already in clinical use [5]. Carcinoembryonic antigens (CEA), cancer antigen 125 (CA-125), and prostate-specific antigen (PSA) are quantitatively assessed for the detection and screening of colorectal [6], ovarian [7], and prostate tumors [8], respectively. However, these tumor-associated antigens have shown low specificity for differentiating the cancer patients from healthy individuals [9]. Hence, the liquid biopsy platforms based on these tumor antigens may result in false negative diagnoses [10].

Circulating tumor cells (CTCs) and exosomes have emerged as potential liquid biopsy biomarkers for determining the histological features, aggressiveness and metastatic potential of the tumor [11, 12]. CTCs are cells that have detached from the primary tumor and circulate through the blood stream [13]. A myriad of technologies have been developed to enrich CTCs from human blood samples, including the method utilizing cancer-targeting capture agents (antibody/peptide-based isolation) and the method employing differences in physical properties between CTCs and other blood components (label-free isolation) [14]. Despite vigorous efforts in developing more sensitive and effective CTC isolation platforms, analyzing tumor markers from CTCs is still challenging due to their low abundance in the blood [15].

In contrast, exosomes, the endosomal-derived vesicles, have obtained great attentions due to their abundance in the body fluid and high stability under varying conditions [16, 17]. Exosomes have been known to involve in cell-to-cell communication that their role in cancer development, progression, and metastasis has been investigated extensively [18, 19]. One of the biggest challenges that needs to be addressed for using exosomes as a tumor biomarker is to minimize their loss during a series of purification processes [17]. Multiple purification steps are required to enrich exosomes from a large spectrum of cellular debris, which eventually decreases the overall yield of exosomes. Another challenge for the exosome-based cancer screening platforms is that these vesicles are not only secreted from the cancerous cells, but also released from most of the mammalian cells [20]. Thus, separating cancer-associated exosomes from the exosomes of non-cancerous origin requires additional processing beyond their enrichment.

Herein, we propose a novel cancer screening method which could detect the tumor-associated expressions in duplicates, by assessing both CTCs and CTC-derived EVs from a single sample. Our team previously developed a highly-sensitive, viable CTC filtration device made of monofilament polyester, which we named fabric filter [21–23]. Cancer cells captured on the fabric filters are subsequently treated with cytochalasin B (CB), in order to release large number of EVs from the cell surface. Precisely controlling the concentration and treatment time of CB enables cells to secrete large number of vesicles from their surface, without affecting the expression levels of cancerous proteins. Multi-modal analysis of CTCs and CTC-derived EVs could provide a potential to overcome the limitations of both CTC and exosome-based liquid biopsy platforms, which suffer from low sensitivity and specificity, respectively. The results presented in this paper provide an idea for the development of a novel cancer screening platform which could eventually enhance the detection sensitivity and accuracy of the current liquid biopsy systems.

## Materials and methods

### Cell preparation and cytochalasin B treatment

Human breast cancer cell line, MCF-7, was treated with cytochalasin B (CB, Tocris Biosciences, MO) at varying concentration of 0, 5, 10, 15, and 20 μg/mL and for varying incubation time of 0.5, 3, and 24 h, respectively. Cell viability was measured using two-colored live/dead cell viability assay kit (Life Technology, CA) and AlamarBlue (Thermo Scientific, IL) as described elsewhere [24, 25]. See Additional file 1 for additional details.

### Fabric sheets preparation

Two types of fabric filters were prepared in this study: the fabric filter prototype 1 (P1) and prototype 2 (P2), which were designed by 2/2 and 3/1 twill patterns, respectively, using 20-denier polyester monofilaments [21, 22]. The details for the filter design and manufacturing process could be found in our previous publications [21, 22]. Briefly, the difference in twill structures affected the size of a slot between the neighboring wefts, as P2 showing larger slot width than P1 (~ 12 µm vs. ~ 8 µm) [21, 22]. Throughout the previous studies, we demonstrated that both fabric filters are capable to capture viable CTCs [21–23]. The structure of each prototype was once more confirmed using SEM. Details could be found in Additional file 1. In this study, the fabric filters were further functionalized with collagen type 1 (3.44 mg/mL), to improve the biocompatibility. Enhancement in biocompatibility was validated by quantitatively comparing the viability and adhesive properties of the cancer cells after culturing them on either collagen-coated or non-modified fabric filters.

### Imaging and characterization of extracellular vesicles

The size and morphology of CB-induced EVs were imaged using field emission scanning electron microscope (FE-SEM, SU5000, Hitachi, Japan) with an acceleration voltage of 5 kV. Nanoparticle tracking analysis (NTA) was performed using Nanosight N5300 (Malvern Instruments, UK), in order to obtain the size distribution and the number of vesicles. The amount of proteins and morphology of EVs were further quantified using BCA assay (Thermo Scientific) and SEM imaging, respectively. See Additional file 1 for additional details.

### Immunofluorescence and immunocytochemistry analysis

We compared the expressions of two cancer-related markers, epidermal growth factor receptor (EGFR) or/and epithelial cell adhesion molecule (EpCAM), based on immunocytochemistry (ICC) and immunofluorescence (IF) assays. ICC was performed directly to the CB-treated cells loaded on the fabric filters using the antibody against human EGFR (R&D Systems, MN), as described previously [25]. IF was conducted after retrieving the captured cancer cells from the fabric filters and immobilizing them on the microscope slides. Cells were then stained with both rhodamine-conjugated EGFR and FITC-conjugated EpCAM (R&D Systems). See Additional file 1 for additional details.

### Quantitative real-time polymerase chain reaction

EVs were lysed and cancer-associated genes were identified using quantitative real-time polymerase chain reaction (qRT-PCR), as described in elsewhere [26]. Primer sequences of EGFR (Hs01076078_m1) and EpCAM (Hs00158980_m1) were purchased from Thermo Fisher Scientifics.

## Results and discussion

### The effect of cytochalasin B on cell viability and vesicle secretion

**Fig. 1:**
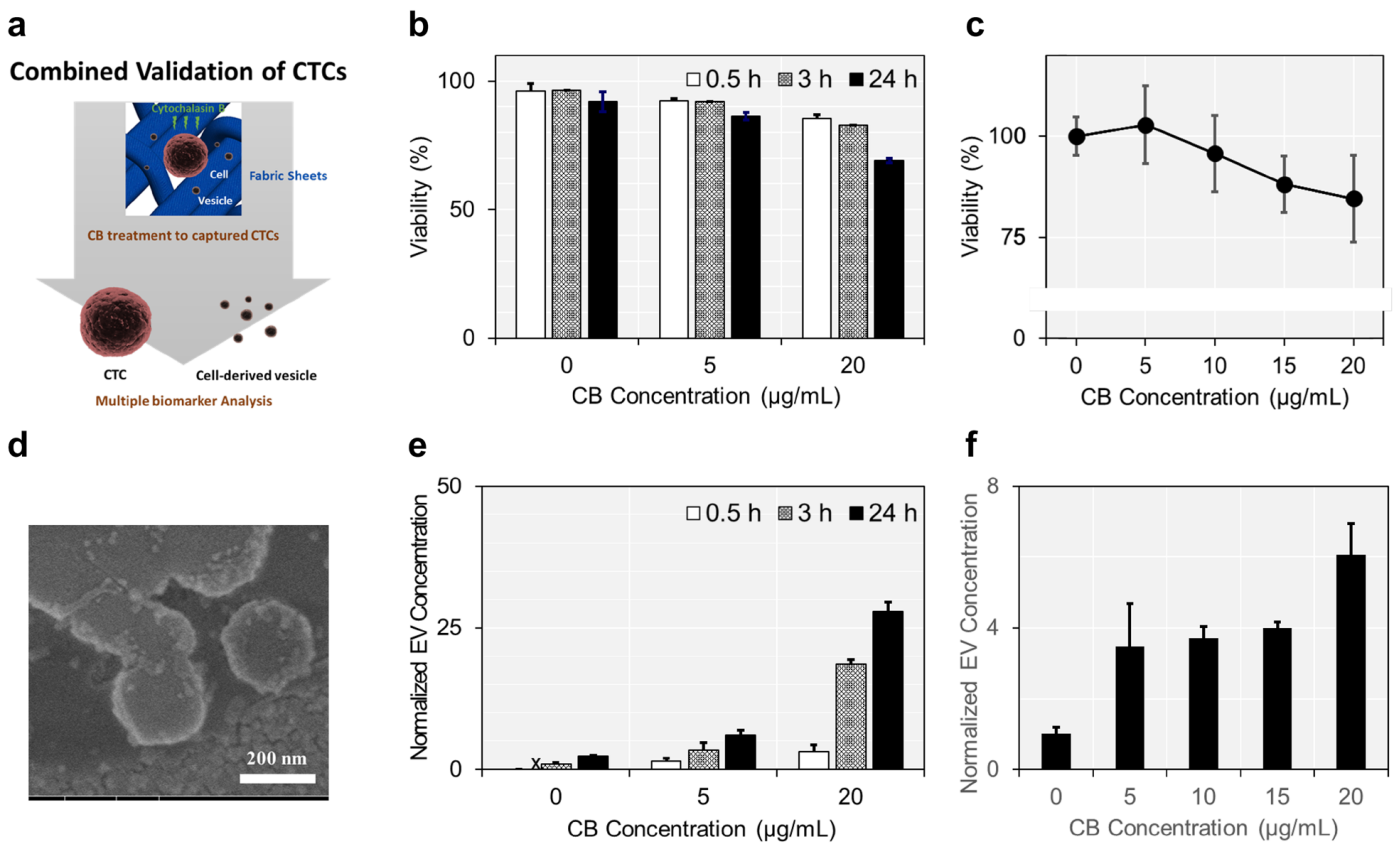
The effect of CB on cell viability and EV secretion: **a** precisely controlling the CB concentration and treatment time enabled multi-modal screening of CTCs. CB treatment promoted cancer cells captured on the fabric filters to secrete large number of EVs with minimal cytotoxic effect. **b** Viability of CB-treated cancer cells were measured using two-colored live/dead assay. **c** Viability of cancer cells were further quantitatively measured in depth, using Alamar blue viability assay after 3 h-CB treatment. No significant cytotoxic effects were found at the CB concentration of ≤ 10 µg/mL and treatment time of ≤ 3 h. **d** SEM was utilized to image the EVs secreted from MCF-7 cancer cells. **e**, **f** CB treatment significantly enhanced the EV secretion. CB increased EV secretion by 3.5-fold at the concentration of 5 μg/mL and there was no further significant increment in EV concentration until it reached 20 μg/mL

Figure 1a illustrates the overall experimental design of the present study. We first determined the optimal CB concentration and treatment time which could promote cells to release large number of vesicles, while having minimal cytotoxic effect. CB has been well-established as a metabolite which could promote cells to release large number of EVs from their surface by involving in cytoskeleton-membrane interaction [27]. These EVs have been exploited in a wide range of biomedical applications, as they are known to reflect the biological characteristics of the parental cells that they have been originated from [28]. For example, CB-induced EVs have been utilized as a receptor for olfactory sensors and a nano carrier for drug delivery systems [29, 30]. However, when CB concentration exceeded certain limit, it strongly inhibited actin polymerization and eventually promoted cell death [31]. Rengan et al. treated CB on lymphocytic cells and verified that CB had remarkable cytotoxic effect when its concentration increased from 1 to 10 μg/mL [32]. It is therefore important to determine the optimal CB concentration and treatment time by balancing its effects on cell viability and vesicle secretion, in order to apply this platform in multi-modal liquid biopsy system based on cell-EV dual screening.

The cells incubated on conventional cell culture flasks were treated with CB at different concentrations and for different time intervals. Cytotoxic effect of CB was investigated using two-colored live/dead staining assay, as demonstrated in Fig. 1b. Cell viability decreased below 90% when the CB concentration exceeded 5 μg/mL or treated over 3 h. Cell viability was assessed more specifically using Alamar blue viability assay after 3 h of CB treatment (Fig. 1c). There were no significant differences in cell viabilities between the cells that have been exposed to CB at the concentration ranging from 0 to 10 μg/mL. However, cell viability reduction was significant when CB concentration exceeded 10 μg/mL, as demonstrated by cells showing 88.1 ± 7.0% (p = 0.031) and 84.6 ± 10.8% (p = 0.057) viabilities at the CB concentration of 15 and 20 μg/mL, respectively. We also found that the cytotoxicity of CB increased 8% when the treatment time increased from 3 to 6 h (Additional file 1: Figure S1). All together, these results indicated that the cytotoxic effect of CB was not significant at the concentration ≤ 10 μg/mL and the treatment time ≤ 3 h.

On the other hand, the number of EVs released from the cells increased exponentially as either CB concentration or treatment time increased (Fig. 1d, e). CB-treated cells secreted 2.5-fold (CB concentration of 5 μg/mL; p = 0.001) and 11.5-fold (CB concentration of 20 μg/mL; p = 0.001) more EVs from the cell surface compared to the non-treated cells after 24 h cell incubation. Notably, there was no significant difference between the size of CB-induced EVs and naturally secreted vesicles (Additional file 1: Figure S2). The effect of CB on EV secretion was analyzed in more depth by fixing its treatment time to 3 h (Fig. 1f). CB increased EV secretion by 3.5-fold at the concentration of 5 μg/mL and there was no further significant increment in EV secretion until the concentration reached 20 μg/mL. Alternatively, we also quantified the amount of vesicular proteins after CB treatment (Additional file 1: Figure S3). After 3 h-CB treatment, the amount of vesicular proteins increased by 2.0-fold, compared to that obtained from the first 0.5 h treatment (p = 0.004). The amount of vesicular proteins increased as the treatment time further increased to 24 h, but the difference was not prominent compared to its initial 3 h-treatment. Based on these findings, we decided to treat CB at the concentration of 5 μg/mL for 3 h, which could produce 3.4-fold more EVs compared to naturally secreted vesicles while having non-significant cytotoxic effect.

### Collagen-coating enhanced biocompatibility of the fabric filters

**Fig. 2:**
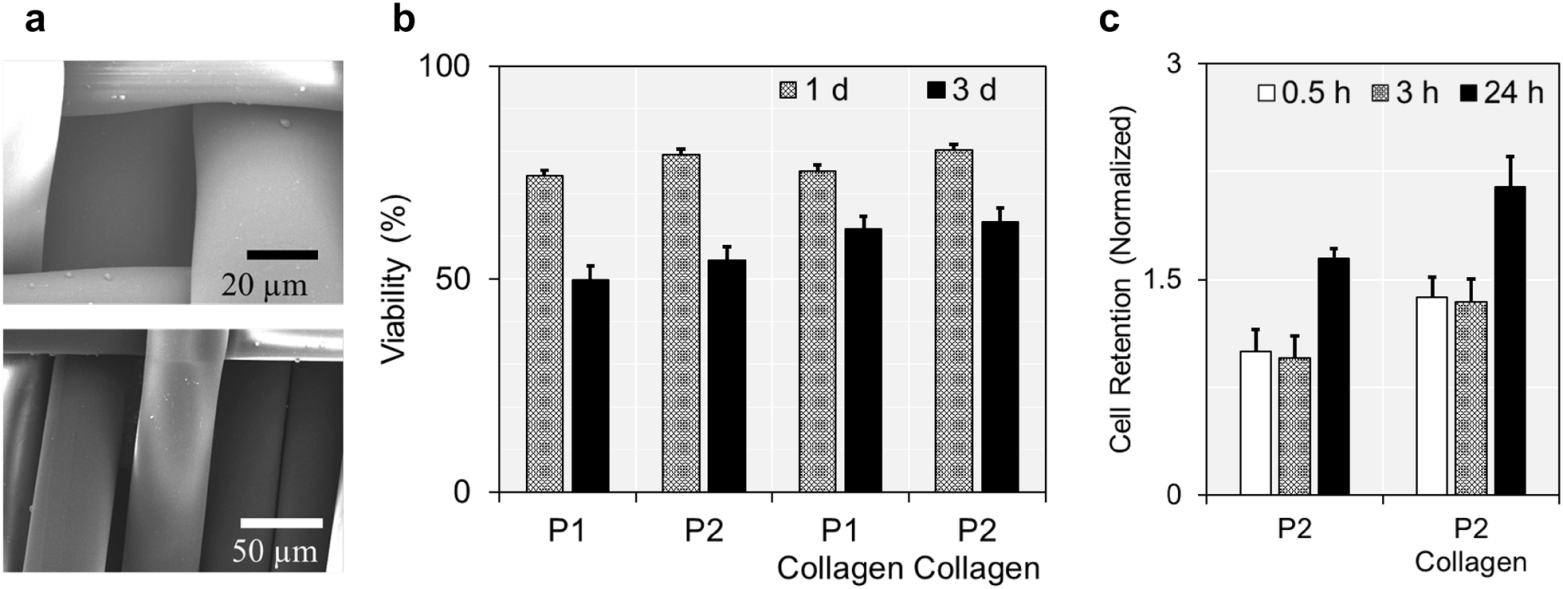
The fabric filters for the multi-modal screening of CTCs: **a** two different fabric filter prototypes, P1 (top) and P2 (bottom), were utilized in this study. The images were obtained using SEM. **b**, **c** The viability of cells loaded on different fabric filter prototypes was quantitatively measured, in order to determine the prototype which could minimize the damage on cells during/after cell capture. Cells on collagen coated P2 showed the highest cell viability among the prototypes

Prior to applying CB treatment on the captured cancer cells, we determined the best configuration among our CTC capture devices, which could minimize the damage on cells after their capture. Note that the cells should be secured properly for the post-capture CTC analysis, since cell viability could influence expression levels of proteins or genes. The viability of cancer cells was measured after isolating them with different types of CTC capture devices, the fabric filter P1 and P2 (Fig. 2a). In our previous studies, we have reported that both fabric filters were capable to isolate viable CTCs with capture efficiency of 70–80% and cell viability of > 80%, at the capture flow rate of 5 mL/h [21–23]. The details are provided in our previous publications. In this study, we found that over 74% of cells were still viable after 24 h-cell culture on the fabric filters (Fig. 2b and Additional file 1: Figure S4). However, this drastically decreased to ~ 50% when the cells were further cultured on the filters for 48 h.

We coated the fabric filters with Collagen type I, a hydrogel which helps cells adhere to the surface and maintain their structure [33]. As expected, the viability of cancer cells was significantly higher on a collagen-coated filter, compared to those on a non-modified filter. The cells on collagen-coated filters showed ~ 1.2-fold higher cell viability than those on non-modified filters (50.1 ± 2.8% vs. 61.5 ± 2.3%; p < 0.001 for P1 and 53.7 ± 2.4% vs. 63.0 ± 4.2%; p = 0.006 for P2; 72 h after cell capture). The collagen-coated filters also achieved higher cell adhesion compared to the bare fabric filters, as demonstrated by collagen-coated filters showing 1.37-fold (p = 0.003), 1.41-fold (p = 0.004), and 1.30-fold (p = 0.005) more number of cells remaining on the surface after washing the filters with PBS solution (Fig. 2c). These results were all indicative of collagen treatment facilitating more cancer cells to maintain viability on the fabric filters.

Another interesting results we found were that the viability of cancer cells cultured on P2 was always higher than that on P1. The smaller slot width of P1 was effective on capturing tumor cells with higher sensitivity compared to P2, which we have demonstrated from our previous study [21]. But conversely, more amount of force could be applied on the cancer cells when capturing with P1, which would eventually induce more cell death, compared to P2. Although there was only a slight difference and the results were statistically less relevant, our previous studies demonstrated that the cells captured on P2 were more viable than those captured on P1, when the cell viability was measured immediately after their capture (90.3 ± 1.4% vs. 85.7 ± 5.7%) [21, 22]. Collagen treatment or further cell incubation did not make any changes, as the cells captured on P2 always exhibited higher viability than those on P1. Despite both filters demonstrating high performance on keeping cells viable during/after cell capture, we chose collagen-coated P2 prototype as a representative CTC filter model for this study, which had the highest capability in maintaining cell viability.

### Cytochalasin B-treated cancer cells still express tumor-specific markers

**Fig. 3:**
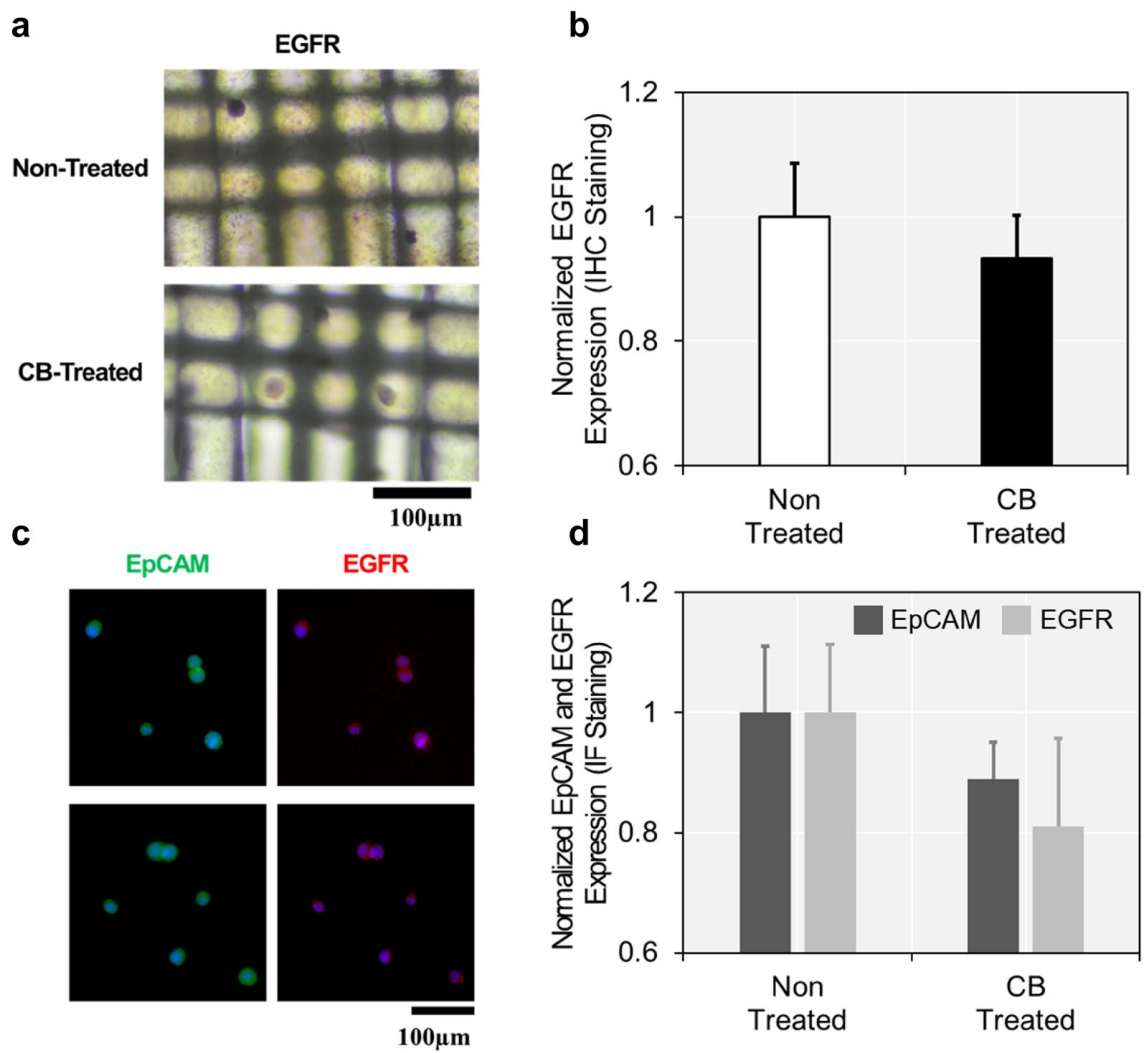
Expression of tumor-associated surface proteins on the cancer cells after CB treatment: **a**, **b** IHC staining was conducted directly on the cells captured on a fabric filter; **c**, **d** IF staining was conducted after releasing the cells from the filter. No significant differences were found in expression levels of EpCAM and EGFR, between CB-treated and non-treated cells

We quantitatively examined and compared the expression levels of two tumor-associated markers, EpCAM and EGFR, before and after treating CB to the cancer cells on the fabric filters. Human epithelial breast cancer cell line, MCF-7, is well known to exhibit high EpCAM and moderate EGFR expressions [34]. We first assessed the EGFR expression based on ICC analysis, immediately after treating CB to the cells loaded on the fabric filters (Fig. 3a, b). EGFR expression was slightly affected by CB, but the changes were not prominent (6.7% decreased; p = 0.057). This result revealed that EGFR proteins were still sufficiently presented on the surface of CB-treated MCF-7 cells.

We extended our study by measuring both EpCAM and EGFR expressions on CB-treated cells based on IF staining. CB-treated cells were trypsinized and released from the fabric filters, prior to IF staining. The effect of CB on expression level of tumor-associated surface proteins was investigated using IF staining, as described in Fig. 3c, d. CB did not affect expression of both EpCAM and EGFR, as there were no significant differences in expression levels of the proteins between CB-treated and non-treated cells. Particularly, EpCAM and EGFR expressions decreased 11.0% (p = 0.132) and 19.9% (p = 0.132), respectively, but were statistically irrelevant. These results confirmed that the CTCs captured on fabric filters could be still utilized as a tumor biomarker, even after CB treatment.

### Cytochalasin B-induced extracellular vesicles express tumor-associated genes

**Fig. 4:**
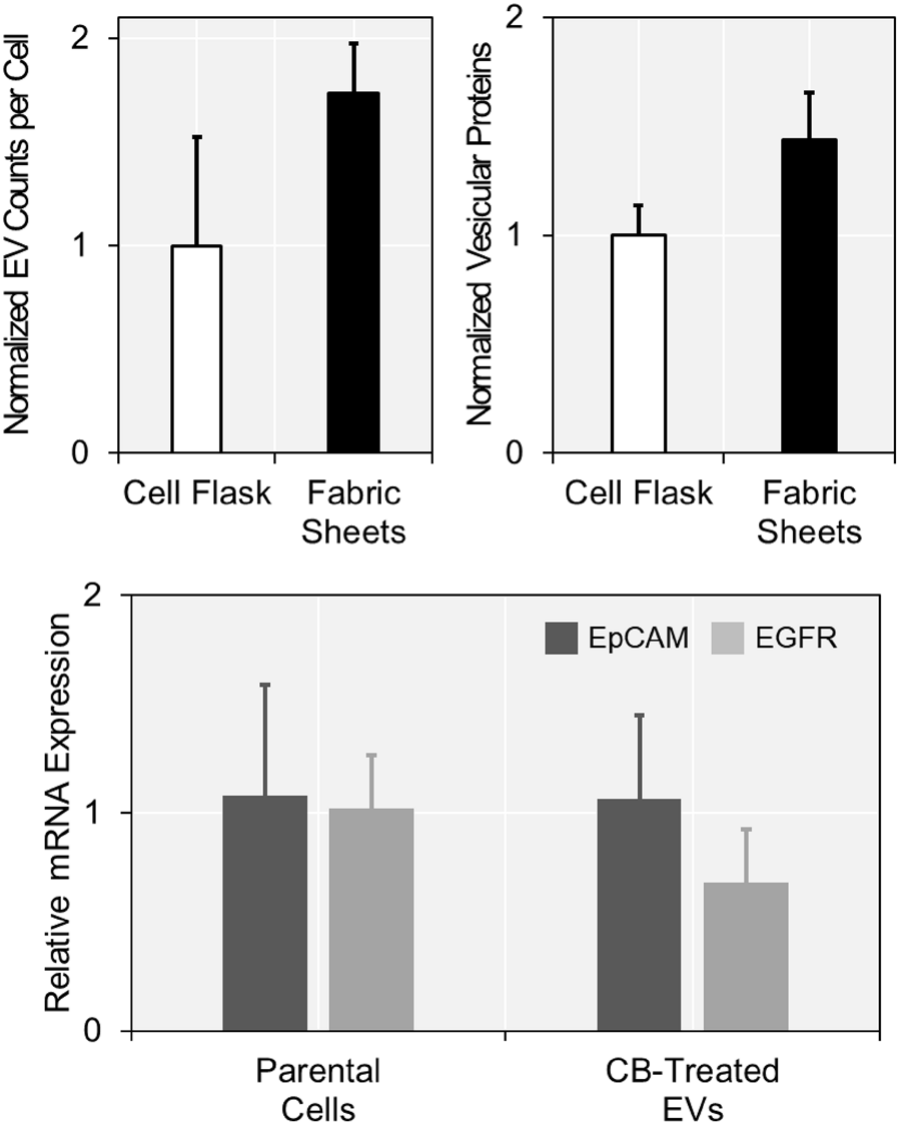
EVs obtained from cells captured on the fabric filters: **a**, **b** the cells on the fabric filters produced more EVs and vesicular proteins, compared to those on the cell culture flasks. **c** EpCAM and EGFR mRNA expressions were detected from CB-induced EVs as well as their parental cells

We treated CB to the cells captured on the fabric filters and compared the number of EVs with the case when CB was treated to the cells on the conventional cell culture flasks (Fig. 4a, b). We hypothesized that the non-specific binding of EVs would decrease on the fabric filters, owing to their multi-pore structures. As expected, the number of EVs produced from the cells on the fabric filters highly exceeded the number of EVs obtained from the cells kept on the conventional cell culture flasks. Cells on the fabric filters produced 1.73-fold (p = 0.011) more EVs and 1.44-fold (p = 0.022) more vesicular proteins, compared to those on the cell culture flasks. The previous studies showed that the CB-induced EVs could retain membrane proteins of the parental cells [35, 36]. Thus, it could be possible for EVs to bind on the cell culture flasks, which is designed to keep cells attached. However, multi-pore structure of the fabric sheets reduced the non-specific binding of EVs, resulting in higher EV yield compared to the conventional cell culture surfaces.

We performed qRT-PCR to verify whether these EVs would reflect the biological status of the parental cells. EpCAM expression of CB-induced EVs did not show any difference with the parental cells (p = 0.969), as described in Fig. 4c. In case of EGFR mRNA expression, expression in EVs decreased ~ 30% compared to that in their parental cells, but the results were statistically irrelevant (p = 0.165). These findings implied that the CB-induced EVs could also be employed as the secondary biomarker for CTC-based liquid biopsy platforms.

Until now, we proved that a larger number of cell-derived EVs could be obtained by simply treating CB to the cells captured on the fabric filters. At the optimized CB treatment condition (CB concentration of 5 μg/mL and treatment time of 3 h), approximately (9.8 ± 1.3) × 10^2^ EVs were released from a single cell per an hour. Considering that CTCs were presented in extremely low concentration, analyzing EVs derived from these rare cells might be a challenge. Total amount of tumor-related proteins or nucleic acids in EVs might not reach the detection limit. However, recent advances in nanobiotechnologies could provide solutions to these challenges. Several methods were proposed to capture and detect tumor-related vesicles, without lysing them [37–39]. Some of these devices were even capable to analyze the vesicles at a single vesicle level. Combining our CTC validation approach with these novel vesicle-screening techniques would enable more reliable tumor screening.

## Conclusion

In this study, we demonstrated a novel CTC-based cancer screening method which could detect tumor-specific expressions simultaneously from a single sample by measuring: (1) surface protein expressions from the captured cancer cells and (2) tumor-associated gene expressions from EVs secreted from the captured cancer cells via CB treatment. CB treatment produced large number of EVs from the cell surface, while the cytotoxic effect of CB was not significant at the concentration of 5 µg/mL and treatment time of 3 h. CB was treated on the cells captured on our previously developed CTC capture device, the fabric filter, after functionalizing its surface with a type 1 collagen. CB produced approximately 9.8 × 10^2^ EVs from a single cell per an hour on the fabric filters, which was 1.73-fold higher than the number of EVs obtained by the cells in conventional T flasks. Most importantly, tumor-associated expressions were detected from both cells and EVs. CB-treated MCF-7 cells exhibited 80–90% expression levels of EpCAM and EGFR proteins on their surface, while CB-induced EVs also overexpressed these tumor-associated genes. Cancer screening based on both CTCs and CTC-derived EVs could potentially bring up highly reliable liquid biopsy platform for more accurate tumor diagnosis and effective anticancer therapy.

## Supplementary information

**Additional file 1.** Materials and methods, **Figure S1**. viability of the cancer cells depending on the treatment time at a CB concentration of 5 μg/mL. ** Figure S2**. the size distribuiton of  CB-induced EVs, **Figure S3**. Amount of vesicular proteins after CB treatment, and **Figure S4**. two-colored live/dead assay for comparing viability of the cancer cells capture on fabric filters.

## Data Availability

All data generated or analyzed during this study are included in this published article and its additional file.

## Abbreviations

CB: cytochalasin B
EVs: extracellular vesicles
CTC: circulating tumor cell
CEA: carcinoembryonic antigens
CA-125: cancer antigen 125
PSA: prostate-specific antigen
FE-SEM: field emission scanning electron microscope
NTA: nanoparticle tracking analysis
EGFR: epidermal growth factor receptor
EpCAM: epithelial cell adhesion molecule
ICC: immunocytochemistry
IF: immunofluorescence
qRT-PCR: quantitative real-time polymerase chain reaction
