# Supplementary material for: Multi-modal liquid biopsy platform for cancer screening: screening both cancer-associated rare cells and cancer cell-derived vesicles on the fabric filters for a reliable liquid biopsy analysis
Source: Nano Converg. 2019 Nov 15;6:39. doi: 10.1186/s40580-019-0204-3 (PMC6856233; doi:10.1186/s40580-019-0204-3)
Supplement: Supplementary file 1 — Additional file 1. Materials and methods, Figure S1. viability of the cancer cells depending on the treatment time at a CB concentration of 5 μg/mL. Figure S2. the size distribuiton of CB-induced EVs, Figure S3. Amount of vesicular proteins after CB treatment, and Figure S4. two-colored live/dead assay for comparing viability of the cancer cells capture on fabric filters. [file 40580_2019_204_MOESM1_ESM.pdf]

Additional File

Multi-modal Liquid Biopsy Platform for Cancer Screening: Screening both cancer-associated rare cells and cancer cell-derived vesicles on the fabric filters for the reliable liquid biopsy analysis

Jiyoon Bu[1,‡,σ], Jae-Eul Shim[1,‡], Tae Hee Lee[1,‡,§], Young-Ho Cho[1,*]

[1] Cell Bench Research Center, Korea Advanced Institute of Science and Technology (KAIST), Daejeon, Republic of Korea

‡ These authors contributed equally to this work

σ Current address: School of Pharmacy, University of Wisconsin-Madison, Madison, WI

§ Current address: Department of Senior Healthcare, BK21 plus program, Graduated School, Eulji University, Republic of Korea

*Corresponding Author: nanosys@kaist.ac.kr, 291 Daehak-ro, Yuseong-gu, Daejeon 34141, Republic of Korea

Key words: Circulating tumor cells; Extracellular vesicles; Cytochalasin B; Polyester Fabric; Liquid Biopsy; Combined validation

Materials and Methods

Cell Preparation

Human breast cancer cell line, MCF-7, was obtained from Korean Cell Line Bank (Seoul, South Korea). Cells were stored in liquid nitrogen in aliquots at a passage number below 10. Every experiment was performed after 3-5 passages post thaw. Cells were grown continuously as a monolayer in T25 culture flasks in RPMI 1640 media supplemented with 10 % (v/v) fetal bovine serum (FBS; Gibco) and 1 % (v/v) penicillin-streptomycin (Invitrogen). The cells were kept in humidified condition at 37 °C with 5% CO_2 and incubated for ~72 h before they reached at 80% confluency. Cells were gently harvested and resuspended to RPMI media, having cell density of 5×10^5 cells per/flask. Cells were incubated further 24 h before CB treatment.

Cytochalasin B Treatment

CB was diluted with 4 mL serum-free media at the concentration of 0, 5, 10, 15, and 20 μg mL^{-1}. Cells were treated with CB for 0.5, 3, or 24 h in humidified condition at 37 °C with 300 rpm agitation. CB-induced EVs were then collected by series of centrifugations. First centrifugation was conducted at 1,000 g for 10 min, in order to remove the cells and large debris. EVs were then further purified by spinning the sample at 14,000 g for 1 h and removing the top 0.5 mL layer. The samples were stored at -70 °C, before it was used for the subsequent experiments.

In case of obtaining EVs from the cells on the fabric filters, we first assembled fabric filters with the filter holders, as described in our previous literatures [1-3]. Previously, we confirmed that our fabric filters could isolate the cancer cells at the capture efficiency of ~70% [1-3]. In this study,

1 mL of cell suspension was gently pipetted through the fabric filter at a cell density of 7.5×10^5 cells/mL. Cells were incubated for two hours on the filter and processed at the flow rate of 5 mL/h for an hour. The number of cells remained on the filters was measured by subtracting the number of cells released from the filter. Approximately $(5.0 \pm 0.9) \times 10^5$ cells were captured on the filter. Filters were gently removed from the filter holders and placed on Corning® Costar® Ultra-Low Attachment 6-well plate. The cells on the filters were further incubated with complete media for 24 h and subsequently treated with CB at the concentration of 5 μg/mL for 3 h, which is determined as the optimal CB concentration and treatment time.

Cell Viability Measurement after Cytochalasin B Treatment

Viability of the cancer cells was measured using two-colored live/dead viability assay (Invitrogen), following the manufacturer's instructions. Briefly, cells were incubated with live/dead staining solution for 30 min which consists of fluorescein isothiocyanate (FITC)-conjugated calcein acetoxymethyl (calcein AM) and tetramethylrhodamine (TRITC)-conjugated ethidium homodimer (ET). Cells were washed three times with PBS solution and examined through the inverted microscope (Ti-E, Nikon). The number of live and dead cells were counted using MetaMorph software (Molecular Devices) and the cell viability was obtained for each of different conditions.

Field Emission Scanning Electron Microscope (FE-SEM) Imaging

The EVs collected by CB treatment (at the optimal condition) were examined with field emission scanning electron microscope (FE-SEM, SU5000, Hitachi) with an acceleration voltage

of 5 kV. EVs were gently laid above the glass slides and dried at 4°C for 6 h. The slide glass was coated with 3 nm-thick osmium, to minimize the charging effects of the EVs. The images of the EVs were acquired to confirm the size and morphology.

Nanoparticle Tracking Analysis (NTA) and BCA Assay

Nanoparticle tracking analysis (NTA) was conducted using Nanosight N5300 (Malvern Instruments), to obtain the size distribution and the concentration of CB-induced EVs. 200 μL of the sample was loaded on the center of the plate, which was located above the laser module. The module was gently mounted on the main instrument and the size distribution of EVs was visualized by tracking the movements of individual particles. Data processing was done by NanoSight N5300 Control Software.

Total amount of protein for each of different CB concentration and incubation time was measured by extracting from RIPA buffer and protease inhibitors mixture for 15 min. Micro BCA protein assay (Thermo scientific) was used to calculate total amount of proteins in EVs, following the manufacturer's instruction.

Immunocytochemistry Analysis

EGFR expressions were examined based on immunocytochemistry, to verify that cancer-related markers were still capable to be observed after CB treatment. The cells on the fabric sheets were fixed and permeabilized as described in the previous section. Endogenous peroxidase was blocked by incubating the filters with 1 % H_2O_2 in methanol for 30 min at the room temperature.

The filters were rehydrated and washed with PBS solution for three times. The cells were incubated with primary antibody EGFR (1:300 diluted, DAKO) for 1 h and washed with PBS solution. Cells were then reacted with EnVisio FLEX/HRP (DAKO) for 40 min, rinsed again with PBS solution, and incubated in DAB reagent (3,3-diaminobenzidine tetrahydrochloride) (DAKO) for 10 min at the room temperature, followed by another PBS washing. Modified Mayer's hematoxylin (Thermo scientific, MA, USA) was treated for 3 min and rinsed with deionized water (three times, 3 min respectively). The filters were kept in 6-well cell culture plate mounted above the inverted microscope (Carl Zeiss) and the EGFR expressions were measured by comparing the intensity of the cells, before and after CB treatment.

Immunofluorescence Analysis

We compared the signals of two cancer-related markers, EGFR and EpCAM, to verify the effect of CB treatment on surface protein expression. The cells on the fabric filters were fixed with 4 % paraformaldehyde (PFA, Santa Cruz Biotechnology) for 15 min and rinsed with PBS solution for 5 min after CB treatment. Cells were subsequently exposed to PBS solution with 3 % bovine serum albumin (BSA, Sigma chimicals) for 30 min to prevent the non-specific bindings. Cells were then stained with 4',6-diamidino-2-phenylindole (DAPI, 6: 1,000 diluted in PBS), FITC-conjugated anti-EpCAM (5 μg mL^{-1} in PBS solution) and Rhodamine-conjugated anti-EGFR (1: 5 μg mL^{-1} in PBS solution). Expression levels of two cancer-related surface markers were compared before and after CB treatment.

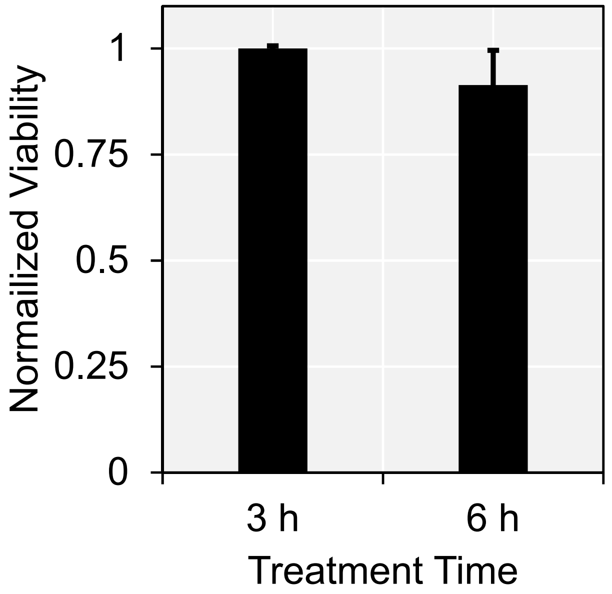

Figure S1. The viability of cancer cells were further quantified using alamar blue viability assay after CB treatment at concentration of 5 µg/mL.

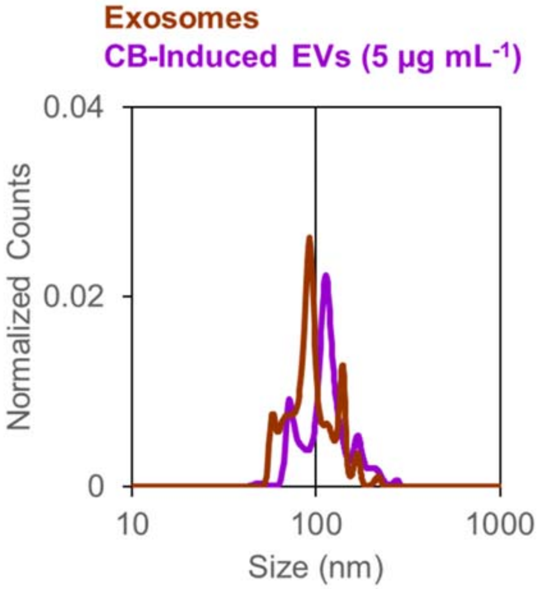

Figure S2. The size distribution of naturally secreted exosomes and CB-induced EVs at CB concentration of 5 μg mL^{-1}.

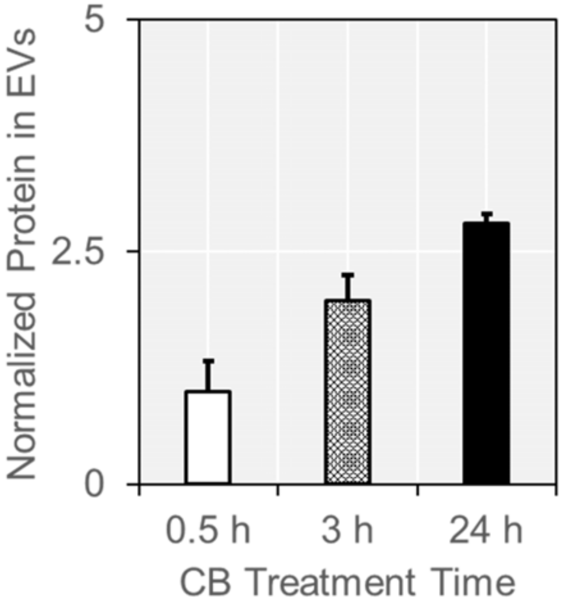

Figure S3. Amount of vesicular proteins depending on CB treatment time at a CB concentration of 5 μg mL^{-1}.

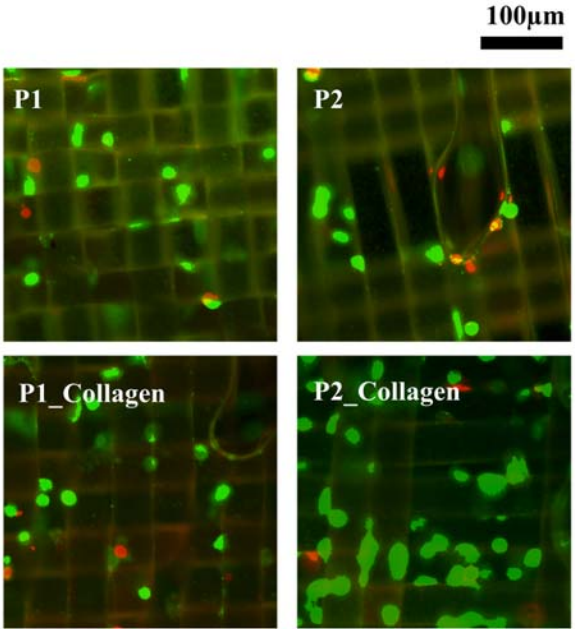

Figure S4. Two-colored live/dead assay for comparing the viability of captured cancer cells, depending on the fabric filter prototype.

References

1. J. Bu, Y.-H. Cho, S.-W. Han, RSC Advances. **7,** 49684-93 (2017)
2. J. Bu, Y.T. Kang, Y.S. Lee, J. Kim, Y.H. Cho, B.I. Moon, Biosens Bioelectron. **91,** 747-55 (2017)
3. J. Bu, Y.J. Kim, Y.T. Kang, T.H. Lee, J. Kim, Y.H. Cho, S.W. Han, Biomaterials. **125,** 1-11 (2017)
